# Measuring the impact of a health information exchange intervention on provider-based notifiable disease reporting using mixed methods: a study protocol

**DOI:** 10.1186/1472-6947-13-121

**Published:** 2013-10-30

**Authors:** Brian E Dixon, Shaun J Grannis, Debra Revere

**Affiliations:** 1 Indiana University, School of Informatics and Computing, Indianapolis, IN, USA; 2 Regenstrief Institute, Center for Biomedical Informatics, Indianapolis, IN, USA; 3 Department of Veterans Affairs, Health Services Research & Development Service, Center for Implementing Evidence-Based Practice, Indianapolis, IN, USA; 4 Indiana University, School of Medicine, Indianapolis, IN, USA; 5 University of Washington, School of Public Health, Seattle, WA, USA

**Keywords:** Evaluation, Health information exchange, Mixed methods, Public health reporting

## Abstract

**Background:**

Health information exchange (HIE) is the electronic sharing of data and information between clinical care and public health entities. Previous research has shown that using HIE to electronically report laboratory results to public health can improve surveillance practice, yet there has been little utilization of HIE for improving provider-based disease reporting. This article describes a study protocol that uses mixed methods to evaluate an intervention to electronically pre-populate provider-based notifiable disease case reporting forms with clinical, laboratory and patient data available through an operational HIE. The evaluation seeks to: (1) identify barriers and facilitators to implementation, adoption and utilization of the intervention; (2) measure impacts on workflow, provider awareness, and end-user satisfaction; and (3) describe the contextual factors that impact the effectiveness of the intervention within heterogeneous clinical settings and the HIE.

**Methods/Design:**

The intervention will be implemented over a staggered schedule in one of the largest and oldest HIE infrastructures in the U.S., the Indiana Network for Patient Care. Evaluation will be conducted utilizing a concurrent design mixed methods framework in which qualitative methods are embedded within the quantitative methods. Quantitative data will include reporting rates, timeliness and burden and report completeness and accuracy, analyzed using interrupted time-series and other pre-post comparisons. Qualitative data regarding pre-post provider perceptions of report completeness, accuracy, and timeliness, reporting burden, data quality, benefits, utility, adoption, utilization and impact on reporting workflow will be collected using semi-structured interviews and open-ended survey items. Data will be triangulated to find convergence or agreement by cross-validating results to produce a contextualized portrayal of the facilitators and barriers to implementation and use of the intervention.

**Discussion:**

By applying mixed research methods and measuring context, facilitators and barriers, and individual, organizational and data quality factors that may impact adoption and utilization of the intervention, we will document whether and how the intervention streamlines provider-based manual reporting workflows, lowers barriers to reporting, increases data completeness, improves reporting timeliness and captures a greater portion of communicable disease burden in the community.

## Background

Health information exchange (HIE) is the capacity to electronically transfer and share health information among health care-related stakeholders and organizations such as clinics, laboratories, payers, hospitals, pharmacies and public health [1]. HIEs promise to reduce health care costs [2], improve patient safety [3], and provide access to more timely surveillance data for public health organizations [4]; however the success of HIE implementations and their system improvements can depend on the context in which data flow and clinical care workflow are integrated. For example, an investigation of one HIE’s impact on emergency department (ED) charges reported a reduction of approximately $26 per encounter (*p* = 0.03) at one hospital with no effect on charges at a second independent hospital in the same HIE [5]. Similarly, in a comparison of HIE utilization among municipalities, Maenpaa et al. (2012) reported a difference in system usage between specialized and primary care providers depending on the numbers of ED visits, laboratory tests, radiology exams and appointments [6]. An investigation of HIE usage in EDs and ambulatory clinics also found that system utilization varied by site, patient population and characteristics, specialization of services, and site policies governing use and administrative access [7]. Studies such as these suggest that outcomes resulting from HIE interactions, access and interventions can be context-sensitive.

An unexplored area is reporting of communicable and infectious diseases, such as pertussis, tuberculosis, salmonella, Chlamydia and Hepatitis C, among others, to public health in an HIE. Most states utilize a dual reporting structure: mandatory case reporting of a disease by providers and mandatory reporting of test results by laboratories to public health authorities. Conventional provider-initiated paper-based reports, transmitted through fax and mail, have been shown to be incomplete, error-prone and untimely. Report completeness ranges from 9 to 99 percent [8]. Highly prevalent diseases like sexually transmitted infections are reported approximately 79 percent of the time and many diseases like pertussis and Lyme disease are reported less than 50 percent of the time [8]. Report timeliness of reporting ranges from one day to three weeks after diagnosis, depending on the disease [9]. In addition, provider reports often lack demographic details that public health workers need, requiring them to perform follow-up calls to get this additional information [10].

In comparison to conventional paper-based reporting, electronic laboratory reporting (ELR) has been successful in delivering more timely laboratory test results to public health [11] and increasing the proportion of notifiable disease reports that are reported to public health [11,12]. However, implementation of ELR can increase or exceed local investigative capacity by significantly increasing the volume of reported cases to public health agencies [13,14] and, like provider reports, ELR can lack clinical and treatment details, such as complete patient demographics, vital signs, pregnancy status or prescribed drugs, needed to fully characterize or prioritize a case for public health purposes [15,16].

Integration of ELR into electronic health record (EHR) systems and HIE networks—i.e., an ELR-EHR-HIE infrastructure—has been shown to connect clinical and public health stakeholders without interrupting existing workflows or adding burden to clinical providers [17]. Given that many states have (or will have in the near future) an infrastructure supporting ELR and HIE [18] and EHRs contain clinical and treatment data often missing from ELR, there is potential to use an integrated ELR-EHR-HIE infrastructure to improve provider-based notifiable disease reporting beyond existing improvements to lab-based reporting.

The “Improving Population Health through Enhanced Targeted Regional Decision Support” study aims to leverage an available ELR-EHR-HIE infrastructure to electronically pre-populate provider-submitted notifiable disease report forms with available clinical, lab and patient data. This intervention has the potential to streamline provider-based reporting workflows, lower barriers to reporting, increase data completeness, improve reporting timeliness and capture a greater portion of communicable disease burden in the community.

We hypothesize clinics that implement the intervention will effectively incorporate the pre-populated reporting form into their workflow, providers will report high satisfaction with the intervention, and that, compared to clinics in which the intervention is not implemented, barriers to reporting will be reduced, timeliness of reporting and completeness of report data fields will improve, and need for providers to provide supplemental or corrected data to public health will be reduced. The implementation of the intervention will be evaluated using mixed methods. The study protocol described will evaluate the implementation of the pre-populated form intervention in the context of an operational HIE.

## Methods/Design

The evaluation-specific objectives are to assess the implementation of the reporting form intervention at the clinic level to: (1) identify barriers and facilitators to implementation, adoption and utilization of the pre-populated reporting form; (2) measure impacts of the tool on workflow, provider awareness, and end-user satisfaction; and (3) describe the contextual factors that impact the effectiveness of the intervention within heterogeneous clinical settings and the HIE.

### Setting

The intervention will be implemented within one of the largest and oldest HIE infrastructures in the U.S., the Indiana Network for Patient Care (INPC). The INPC is anchored by the Regenstrief Medical Record System which collects data from a variety of sources, including hospitals, clinics, pharmacies, and laboratories [19]. Lab results are routinely delivered to ordering physicians using the Regenstrief DOCS4DOCS® software and analyzed as ELR transactions by the Notifiable Condition Detector (NCD) which identifies mandated reportable diseases and notifies local and state public health departments of these laboratory results [20].

### Participants

The study will include outpatient INPC primary care practices operating in urban and rural settings that are part of the INPC, representing multiple health systems and clinics. Clinics that do not use the DOCS4DOCS® software will be excluded.

### Intervention

Two technical interventions will be deployed over a staggered schedule at participating clinics: 1) “standard” pre-populated forms and 2) “enhanced” pre-populated forms. The “standard” forms intervention will use EHR (patient demographics and clinic information) and ELR (notifiable disease test results) data available in the HIE to pre-populate and deliver an electronic version of the official state notifiable disease reporting form to the provider. Providers will be able to review the pre-populated form, add any additional information, and fax completed forms to their local health department. The “enhanced” forms intervention will pre-populate an alternative reporting form with an expanded set of data available in the HIE. For example, the “enhanced” form will not only include test results data for a case of hepatitis B but also corollary results on the patient’s liver enzymes; this is information the health department typically requests from the provider in a follow-up phone call when investigating the reported case of hepatitis. Providers will still be able to review the pre-populated “enhanced” form, add any additional information, and fax completed forms to their local health department. Since deployment is staggered, at any point in time the non-intervention sites can act as natural controls for the intervention sites without the selection bias that is generally present in non-randomized experiments. Therefore, the study protocol is theoretically equivalent in its ability to generate causal evidence to a traditional randomized controlled experiment.

### Research questions

Mixed methods studies require that research questions be linked to and drive the data collection and analysis methods, as well as inform the study design, sample size, sampling, instruments developed and administered, and data analysis techniques [21]. Our primary research questions are:

1. What individual, organizational and data quality factors may act as barriers or facilitators to the successful adoption and utilization of pre-populated reporting forms and enhanced data transaction processing to public health; and

2. What is the relationship of these barriers and facilitators to fostering improvements in provider-based population health reporting workflows, lowering barriers to reporting and case follow-up, increasing data completeness, and enabling greater capture of communicable disease burden in the community?

### Data collection

Using a concurrent mixed methods design, data collection will be conducted during the three project phases: baseline or pre-implementation; post-implementation of the standard form; and post-implementation of the enhanced form. In each phase, qualitative and quantitative data are collected in tandem as coordinated but independent studies. This design will allow us to triangulate the quantitative results from surveys, time-series, and data quality measures with qualitative interview and open-ended survey results to understand experiences with public health reporting before and after each form implementation. Table 1 summarizes the categories of data collected.

**Table 1:** Summary of study constructs, data collection, analysis approaches and outcomes measurements by method (qualitative, quantitative)

**Evaluation construct**	**Data collected**	**Tool/method**	**Analysis**
Reporting rates	Provider reports to public health	C	ITS
Completeness	Completed/missing provider report data fields	C	ITS
	Comparison of completeness between S & E forms	C	PPC
	Perceptions of completeness of pre-populated forms	S/I	QUAL/DESC
Accuracy	Inaccurate provider report data fields	C	ITS
	Comparison of accuracy between S & E forms	C	PPC
	Perceptions of accuracy of intervention	S/I	QUAL/DESC
Timeliness	Time between patient diagnosis & treatment	C	ITS
	Time between between lab-confirmed diagnosis & report to public health	C	ITS
	Comparison of timeliness between S & E forms	C	PPC
	Perceptions of timeliness of intervention	S/I	QUAL/DESC
Burden	Volume & duration of phone & Fax communications	C	ITS
	Perceptions regarding reporting burden	S/I	QUAL/DESC
Data Quality	Perceptions regarding quality of data in pre-populated reporting forms	S/I	QUAL/DESC
Form Enhancement	Supplementary data & fields of value to public health	FG	QUAL/PPC
Benefits & Utility	Perceived benefits & utility of intervention	S/I	QUAL/DESC
Adoption & Use	Perceived barriers & facilitators to adopting & using pre-populated report forms	S/I	QUAL/DESC
	Level of acceptance & satisfaction with intervention	S/I	QUAL/DESC
	Perceived ease of operations	S/I	QUAL/DESC
	Acceptability of interface	S/I	QUAL/DESC
Workflow	Public health workflow observations	O	DESC
	Reported impact of intervention on work & information flows	S/I	QUAL/DESC
Context	Clinic demographics	S/I	DESC

KEY.

*C* Census of public health report forms & data fields; *DESC* Descriptive Statistics; *FG* Focus Groups; *I* Semi-Structured Interviews; *ITS* Interrupted Time-Series; *O* Observations; *PPC* Pre-Post Comparison; *QUAL* Qualitative Data Analysis; *S* Provider Surveys.

### Quantitative data collection

The following data will be collected at baseline (retrospective to 12 months prior to introduction of the intervention) and at 6-, 9-, and 12-months after implementation of both form interventions: reporting rates (the number of reports for individual diseases and in aggregate submitted to public health daily); report data completeness (completeness of fields) and accuracy (errors); reporting timeliness (length of time between the laboratory test date and treatment); treatment timeliness (length of time between the laboratory test date and treatment); and reporting burden defined as communication volume (number of phone calls or FAX communications between public health and clinics/providers or laboratories) and duration (total number of minutes) measured at the level of phone call.

### Qualitative data collection

Semi-structured interviews will be conducted with representative clinical and public health workers will collect qualitative data regarding provider and public health perceptions of completeness, accuracy, timeliness and burden associated with notifiable disease reporting prior to and after each form intervention. In addition, perceptions regarding data quality, benefits, utility, adoption, utilization and impact on workflow of reporting prior to and after the intervention will be collected during baseline and at 12-months after implementation of each form intervention. Interviews will be digitally audio-taped and transcribed. Public health practitioner input regarding enhancements and supplemental data preferences for the enhanced report form will be captured by convening focus groups to establish consensus on desirable data elements.

### Data analysis

Data analysis methods will be more thoroughly reported in the methods sections of future publications presenting the findings of specific analyses.

### Data analysis (quantitative)

Quantitative analysis will provide measurable evidence of the impacts of the intervention and enable us to establish likely cause and effect. Longitudinal effects of the interventions will be evaluated in aggregate and across covariates on reporting rates, reporting timeliness and treatment timeliness using an interrupted time-series design to test for the intervention effect and the time trend post-intervention. This approach has been used in other decision support and time-series evaluations [22] and has been recommended for multiple baseline time-series designs that involve two or more sites that are repeatedly assessed while an intervention is introduced into one site at a time [23]. Since the effects of the interventions may vary across clinics, stratified regression analyses will also be performed. Following comparative assessment of reporting completeness, accuracy and communication burden, these data will be added to the regression model. Assessment of data completeness, timeliness, and accuracy will involve pre-post comparison of data quality metrics [15].

### Data analysis (qualitative)

Qualitative analysis will provide in-depth context regarding facilitators and barriers to implementation, adoption, benefits and use of the intervention; identify and describe their impacts on HIE individual and organizational processes; and look at the broad range of interconnected processes or causes at play regarding data quality. Analyses will be conducted using qualitative software by experienced coders using the constant comparative method of analysis [24] and utilizing standard approaches to ensure credibility, consistency and robustness of the findings. Transcribed interviews and open-ended survey items will undergo a series of well-established steps to identify emerging themes and trends and, ultimately, build a model to describe the intervention phenomenon in a conceptual form [25]. The process will begin with developing a coding scheme which will be developed from combining concepts derived *a priori* from the conceptual frameworks driving the study and inductively as the analysis proceeds. Content will be grouped into nodes, a codebook will be built, and codes or code combinations will be summarized and stratified by contextual factors such as demographics, respondent role, etc. These summaries will be entered into appropriate data displays that specify interactions between the intervention, its context and its effects as preparation for triangulation.

### Triangulation of quantitative and qualitative data

Data will be triangulated to find convergence or agreement by cross-validating results. The first step will be exploratory to determine most appropriate “transformation” of the data: conversion of quantitative data into narrative data (“qualitized”) or conversion of qualitative data into numerical codes (“quantitized”) that can be represented statistically. There are several approaches to quantifying qualitative data, including enumerating the frequency of themes within a sample, the percentage of themes associated with a given category of respondent, or the percentage of people selecting specific themes. In these approaches the quantified data can be statistically compared to quantitative data collected concurrently but separately. Another strategy for quantifying qualitative data enumerates whether or not qualitative responses included certain codes—i.e., rather than seeking to understand how many times a certain code was provided by each participant or the frequency with which they appeared, the presence or absence of each code for each participant is quantified into dichotomous variables 0 or 1 based on absence or presence of each coded response. We will determine the most appropriate approach after reviewing the descriptive analysis. Depending on which approach is used, this mixed data will be correlated (quantitative data correlated with qualitized data or vice versa) [26]. Guided by the nature of the data collected, quantitative and qualitative data may be collated to create new or consolidated variables or data sets in order to further compare and integrate data.

Once transformed, we will calculate simple correlations, stratify codes by provider type, demographics, geographic location or organization attributes or look at similarity among respondents. This process will allow us to identify areas in which findings agree (convergence), contradict (discrepant or dissonant) or deepen understanding on the same issue (complementarity) [27]. We anticipate that the results will allow us to identify, analyze and explain social, behavioral and environmental similarities and differences between HIE settings, provider types and perceptions across pre- and post-implementation time units that will describe identified barriers and facilitators to the intervention.

### Limitations and biases

There are several limitations to our proposed work. First, some clinical sites may be more open to recruitment and enrollment in the study and thus introduce a bias in our sample. For example, some sites may serve patient populations that are more likely to require notifiable disease reporting (for example, a women’s clinic with a high Chlamydia reporting history) and thus be more incentivized to participate. It is possible given use of an interrupted time-series design, that confounding may occur due to covariates that may change over time. The INPC is a growing HIE so it is possible that policy, governance, legal mandates or information technology changes could occur during the course of this study that may impact data collection or introduce additional confounding issues. This HIE includes an academic affiliation and medical training program so providers may rotate through training sites at different stages of the intervention which may introduce confounding. Introduction and adoption of the intervention may be more rapid in some settings (small ambulatory clinic) than others (large hospital) as the workplace may accommodate or adjust to the intervention more easily or require administrative protocols to support the intervention. It is also possible that the staggered implementation of the intervention may complicate quality control of data collection. Also, given data collection covers only one baseline year and a maximum of two years post-intervention, depending on site, this short time frame may limit ability for analysis to account for seasonal trends in reporting. Our findings may also be limited by the context in which this study is conducted. The INPC is one of the most advanced HIEs in the country; therefore, our results may have limited generalizability. However, we believe our emphasis on context and by clearly documenting the characteristics of the sites in which the intervention, our findings could inform implementation other HIE settings. Given current mandates to build systems and infrastructures for ELR and HIE in communities where it is absent, expand efforts in communities where ELR is already occurring, and requirement for eligible hospitals to routinely transmit reportable laboratory results to a public health agency, our findings may provide insights and inform roadmaps for more nascent HIEs to move forward towards better notifiable disease surveillance.

### Ethical considerations

The project received approval by the Institutional Review Board of Indiana University with a concurrent Institutional Review Board deferral from the University of Washington to Indiana University. Informed consent will be obtained from all participants and confidentiality will be ensured. All data will be stored according to the rules of the research ethics committee. Because this study does not meet ICMJE guidelines it has not been registered in a publicly accessible registry.

## Discussion

Context is recognized as a critical element for understanding the effects of intervention components individually and in combination [28]; appropriately generalizing findings across multiple sites [29]; and accelerating the process of translating research into practice [30]. Context includes the characteristics of an organization and its environment, e.g. size, organizational structure, personnel, training, governance, financial factors, leadership, legal mandates, communication flows, which can influence the implementation and effectiveness of an intervention. However, the use of conventional quantitative research methods alone often limit detailed understanding of the phenomena under study, ignore the context of the problem or intervention, minimize the impact of the changing nature of the study subject and/or its context, and exclude outliers in preference to generating implications from the mean [31]. Sensitivity to context and “context-heterogeneity”—i.e., how differences in context change, shape and are shaped by the phenomena under study—is one of the values of qualitative methods.

Mixed or multi-methods research, which integrates quantitative and qualitative data collection and analysis in a single study or a program of inquiry, provides methodologies for measuring the effects of an intervention within complex, growing and evolving contexts while also exploring the context of the intervention itself [32,33]. In the HIE setting, employing mixed methods could contribute to better understanding of the contextual issues that influence the potential quality, safety and efficiency outcomes associated with HIE implementations [34].

Combining quantitative and qualitative methods in our evaluation of a complex HIE intervention has two potential benefits: complementarity and multiplicativeness, i.e., the methods may illuminate different aspects of the intervention impacts and the combination of the two methods may provide greater insight than either method individually [35]. By measuring context, facilitators and barriers, and individual, organizational and data quality factors that may impact adoption and utilization of the intervention, we will document whether and how the intervention streamlines provider-based manual reporting workflows, lowers barriers to reporting, increases data completeness, improves reporting timeliness and captures a greater portion of communicable disease burden in the community. We believe that our approach demonstrates a multi-faceted and integrative research “attitude” towards the problems we are concerned with, the insights we are seeking, the comprehensive ways in which we desire to generate knowledge, and the creative and scientific curiosity required to rigorously tackle an interdisciplinary investigation.

## Conclusion

HIEs are rapidly expanding in the U.S., fueled by national policies that are investing in the integration of ELR, EHR, and other information systems. Yet only one-third of public health departments are engaged in local efforts to electronically exchange data between clinical and public health [36]. Our study not only presents the opportunity to study the evolving public health infrastructure with integration of ELR-EHR-HIE technologies but also the use of mixed methods to better understand the context in which HIE and HIE-based interventions are implemented. To our knowledge, this approach is unique as we were unable to identify previous articles that detail a systematic and rigorous application of mixed research methods to the evaluation of HIE. By testing the feasibility of using mixed methods to study a complex informatics intervention in a complex health care setting, we believe our approach extends prior work in informatics that calls for incremental evaluation of maturing HIE models [34,37]. Similar studies of HIE and public health informatics interventions should consider leveraging the combination of quantitative and qualitative methods to more fully explore, observe, and document the impact of interventions on population outcomes as well as information system adoption and use.

## Abbreviations

EHR: Electronic health record; ELR: Electronic laboratory reporting; ED: Emergency Department; HIE: Health information exchange; INPC: Indiana network for patient care.

## Competing interests

The authors declare that they have no competing interest.

## Authors’ contributions

SG, DR and BD conceived of the study. All authors were involved in study design. DR and BD wrote the draft manuscript. All authors reviewed and participated in revisions of this manuscript. All authors read and approved the final manuscript.

## Pre-publication history

The pre-publication history for this paper can be accessed here:

http://www.biomedcentral.com/1472-6947/13/121/prepub
